# SEPIa, a knowledge-driven algorithm for predicting conformational B-cell epitopes from the amino acid sequence

**DOI:** 10.1186/s12859-017-1528-9

**Published:** 2017-02-10

**Authors:** Georgios A. Dalkas, Marianne Rooman

**Affiliations:** 1 BioModeling, BioInformatics & BioProcesses (3BIO), Université Libre de Bruxelles (ULB), CP 165/61, 50 Roosevelt Ave, 1050 Brussels, Belgium; 2 Interuniversity Institute of Bioinformatics in Brussels, ULB-VUB, CP 263, Triumph Bld, 1050 Brussels, Belgium; 3 Present address: Institute of Mechanical, Process & Energy Engineering, Heriot-Watt University, Edinburgh, EH14 4AS UK

**Keywords:** Immunoinformatics, Machine learning, Antigen-antibody complexes, B-cell epitopes, Statistical potentials, Physicochemical properties, Bioinformatics predictor, β2 adrenergic G-protein-coupled receptor

## Abstract

**Background:**

The identification of immunogenic regions on the surface of antigens, which are able to be recognized by antibodies and to trigger an immune response, is a major challenge for the design of new and effective vaccines. The prediction of such regions through computational immunology techniques is a challenging goal, which will ultimately lead to a drastic limitation of the experimental tests required to validate their efficiency. However, current methods are far from being sufficiently reliable and/or applicable on a large scale.

**Results:**

We developed SEPIa, a B-cell epitope predictor from the protein sequence, which is sufficiently fast to be applicable on a large scale. The originality of SEPIa lies in the combination of two classifiers, a naïve Bayesian and a random forest classifier, through a voting algorithm that exploits the advantages of both. It is based on 13 sequence-based features, whose values in a 9-residue sequence window are compiled to predict the epitope/non-epitope state of the central residue. The features are related to the type of amino acid, its conservation in homologous proteins, and its tendency of being exposed to the solvent, soluble, flexible, and disordered. The highest signal is obtained from statistical amino acid preferences, but all 13 features contribute non-negligibly in the predictor. SEPIa’s average prediction accuracy is limited, with an AUC score (area under the receiver operating characteristic curve) that reaches 0.65 both in 10-fold cross-validation and on an independent test set. It is nevertheless slightly higher than that of other methods evaluated on the same test set.

**Conclusions:**

SEPIa was applied to a test protein whose epitopes are known, human β2 adrenergic G-protein-coupled receptor, with promising results. Although the actual AUC score is rather low, many of the predicted epitopes cluster together and overlap the experimental epitope region. The reasons underlying the limitations of SEPIa and of all other B-cell epitope predictors are discussed.

**Electronic supplementary material:**

The online version of this article (doi:10.1186/s12859-017-1528-9) contains supplementary material, which is available to authorized users.

## Background

The humoral immune system protects the extracellular space from foreign objects like bacteria and viruses. A central role in the immune response is played by antibodies that are secreted by B-cells. These proteins recognize the disease-causing agents and thereby trigger their neutralization. The recognition mechanism involves the binding of antibodies to antigens, which are usually proteins or polysaccharides from the pathogenic substances. Epitopes are the regions of the antigen surface that are bound by the antibodies. The localization and identification of epitopes, which are targeted by specific antibodies and are capable of inducing an efficient immune response, is of utmost importance for the rational design of potential vaccines [1–3].

We focused in this paper on protein antigens. These are classified as linear (or continuous) and conformational (or discontinuous) epitopes, depending on their structure and interaction with antibodies [4]. Specifically, linear epitopes consist of amino acids that are contiguous in the polypeptide chain, while conformational epitopes contain amino acids that are distant along the sequence but spatially close in the native structure. Linear epitopes are often found in peptides and conformational epitopes in proteins.

For over 30 years, computational methods have been developed for facilitating epitope recognition [5]. In the past, the majority of the *in silico* methods were focused on linear epitopes. Most of these approaches are sequence-based and use amino acid-based propensity scales, such as hydrophilicity, solvent accessibility, secondary structure and flexibility; a score derived from the propensity scales is assigned to each residue, and the whole sequence is examined for high-scoring window fragments, which are then predicted as epitopes [6–12]. However, the prediction results of these methods have only marginally better performances than random selections [13]. In the last few years, several groups investigated the combination of multiple amino acid propensity scales to predict linear B-cell epitopes [14–17] with no significant improvement of the prediction success rate. Recently, not only sequence-based, but also structure-based, amino acid features have been used in conjunction with machine learning methods and have been shown to slightly improve the prediction accuracy of linear B-cell epitope predictions [14–23].

Although the large majority of B-cell epitopes are conformational [24], they started to be studied later. Many groups have analyzed various physicochemical, structural, and geometrical features of epitopes in order to determine which of them significantly distinguish epitope from non-epitope antigen residues [25–29] and what are the characteristics of antigen-antibody interfaces compared to other protein-protein interfaces [30–33]. The existing conformational epitope prediction tools were developed by combining such informative attributes, which are based either purely on the sequence, or both on the sequence and the structure [34–39]. More recently, machine-learning techniques have been used to improve the prediction performance of conformational epitopes [40–47].

In this study, we describe SEPIa, a conformational epitope prediction method that requires only the amino acid sequence as input and is based on commonly used features, but also on new ones. It utilizes a meta-learning approach, which combines the predictions obtained with two different classifiers through a voting procedure and yields a single prediction with improved accuracy [48].

## Methods

### Datasets

We constructed a non-redundant data set of 85 of antigen-antibody complexes, noted *S85*, from the Immune Epitope Database (IEDB-3D) [49], which is an updated and extended version of the one we used earlier [29]. To increase the number of antigen sequences used for developing our method, and given that the 3-dimensional (3D) structure is only required for the identification of epitope residues, we considered structures with resolution better than 3 Å, against 2.5 Å in our previous study. The other criteria remained the same: (i) for complexes represented by more than one crystal structure, the 3D structure with the best resolution was chosen; (ii) structures in which the antibody binds the antigen but involves no residues from complementarity determining regions (CDRs) were excluded; (iii) complexes in which the antibody does not contain both the light and heavy chains were discarded; (iv) for structures with more than one complex in one asymmetric unit and no structural difference between them, only one complex was chosen; and (v) to obtain a non-redundant data set, the sequences were pairwise aligned using the ClustalW program [50], and if two sequences had a sequence identity of more than 70%, only one was kept. Note that epitopes from similar antigens were kept if these antigens were in complex with different antibody CDR sequences. With this procedure, antibody–antigen complexes were selected and the corresponding coordinate files were obtained from the Protein Data Bank (PDB) [51].

Two of the antigens of the *S85* dataset have common epitopes, which are not identified as epitopes in all antigen-antibody complexes. We defined the *S83* set that contains all 85 antigen chains of *S85* except these two. The lists of antigens of the *S85* and *S83* sets are given in Additional file 1: Table S1.

To determine the epitopes, we proceeded as in reference [29]. We calculated the solvent accessibility values of the antigen residues without taking the antibody into account (ACC_unbound_), using an in-house program [29], and compared them with the accessibility of antigen residues in the complex (ACC_bound_). All antigen residues with a solvent accessibility variation of 5% at least upon antibody binding (ACC_unbound_ - ACC_bound_ ≥ 5%) were considered as epitope residues. The *S85* set contains 1,667 conformational B-cell epitope residues and 16,780 other residues. The ratio between epitopes and non-epitopes is thus almost exactly 1:10.

An independent dataset of 19 antigen sequences [42], noted *S19*, was used to evaluate the predictor and to compare it with other methods; it has already been used for that purpose in other investigations [42, 45, 52]. These sequences and epitope assignments were taken from the conformational epitope database (CED) [53]. The epitope residues were here not identified on the basis of the 3D structure of the complexes, but rather experimentally, with the help of techniques such as surface plasmon resonance, ELISA and immunoblotting. The ratio between epitope and non-epitope residues in this set is 1:13, with 407 epitope and 5,192 non-epitope residues. The members the *S19* set are listed in Additional file 1: Table S2

The sequences from both datasets *S85* and *S19* were pairwise aligned using the ClustalW server [50]. None showed a sequence identity of more than 70%, which is the identity threshold used for building *S85*. The two datasets may thus be considered as independent.

### Features

We evaluated 14 sequence-derived features, referred to as F1–F14. These are:

#### Amino acid composition

It is well known that certain amino acid types show preferences to be located in epitopes, in non-epitope protein surfaces, or in the protein core [29]. We used here two features related to the amino acid composition: the ratio of the amino acid frequency observed in epitopes and in the remaining antigen surface (referred to as F1), and the ratio of the amino acid frequency in epitopes and in the remaining antigen (F2). These two features were computed on the *S85* dataset. Their values are given in Additional File 1: Table S3.

#### Hydrophilicity

A characteristic closely related to the amino acid composition is the hydrophilicity. Epitopes are known to be enriched in charged and hydrophilic amino acids [29]. We used here the hydrophilicity scale of Hopp and Woods [6] as feature F3.

#### Secondary structure

As epitopes have been shown to be more often located in the loop regions of the antigen [29], the predicted secondary structure was added as feature F4. We used for that purpose the program NetSurfP [54] that provides amino acid propensities for being in an α-helix, β-strand or coil. We also used the program BetaTPred3 [55] that estimates the β-turn propensities in protein sequences, and tested them as epitope feature F14.

### Flexibility

Given that epitopes often involve loop regions, flexibility could be expected to be an informative factor. We used two programs to predict flexibility from sequence, DynaMine [56] (F5) and PredyFlexy [57] (F6), which are based on two different definitions of flexibility. DynaMine predicts the backbone flexibility at the residue level in the form of backbone N-H S^2^ order parameter values; a value of 1 means a rigid conformation, while a value of 0 means highly dynamic. PredyFlexy is instead based on root mean square fluctuations (RMSF) obtained from molecular dynamics simulations.

#### Intrinsically disordered regions

The tendency of protein sequences of being structured or unstructured is another feature that could help distinguishing epitope from non-epitope regions. Two web servers were used to calculate such regions from the amino acid sequence. IUPred [58] (F7) predicts intrinsically disordered regions and ANCHOR [59] (F8) disordered binding regions.

#### Energy-like

A novel feature that we introduce here is based on the statistical potential formalism [60–62] to evaluate the propensity of a residue to be part of an epitope. The first “potential”, Δw_1_, measures the influence of a residue of type s at position j along the sequence on the epitope/non-epitope state u (u = E for epitopes and u = N for non-epitopes) of the residue at position i:

1 $$ {\Delta \mathrm{w}}_1\left({\mathrm{u}}_{\mathrm{i}},{\mathrm{s}}_{\mathrm{j}}\right)=-\mathrm{R}\mathrm{T} \ln \frac{\mathrm{F}\left({\mathrm{u}}_{\mathrm{i}},{\mathrm{s}}_{\mathrm{j}}\right)}{\mathrm{F}\left({\mathrm{u}}_{\mathrm{i}}\right)\mathrm{F}\left({\mathrm{s}}_{\mathrm{j}}\right)}, $$

where F are relative frequencies computed from the learning dataset *S85*, R is the Boltzmann constant, T the absolute temperature taken to be room temperature, and j − w ≤ i ≤ j + w, with w an integer between 0 and 8. The window size I = 2w + 1 is a parameter that will be optimized to get the best prediction performances.

The second “potential”, $\Delta {\mathrm{w}}_2$, measures the influence of two residues of types s and s′ at positions j and k along the sequence on the epitope/non-epitope state u of the residue at position i:

2 $$ {\Delta \mathrm{w}}_2\left({\mathrm{u}}_{\mathrm{i}},{\mathrm{s}}_{\mathrm{j}},\mathrm{s}{\hbox{'}}_{\mathrm{k}}\right)=-\mathrm{RTln}\ \frac{\mathrm{F}\left({\mathrm{u}}_{\mathrm{i}},{\mathrm{s}}_{\mathrm{j}},\mathrm{s}{\hbox{'}}_{\mathrm{k}}\right)}{\mathrm{F}\left({\mathrm{u}}_{\mathrm{i}}\right)\mathrm{F}\left({\mathrm{s}}_{\mathrm{j}},\mathrm{s}{\hbox{'}}_{\mathrm{k}}\right)}, $$

with j − w ≤ i ≤ j + w and k − w ≤ i ≤ k + w.

To correct for sparse data, we applied the usual correction [54–56]:

3 $$ \begin{array}{c}\hfill \frac{\mathrm{F}\left({\mathrm{u}}_{\mathrm{i}},{\mathrm{s}}_{\mathrm{j}}\right)}{\mathrm{F}\left({\mathrm{u}}_{\mathrm{i}}\right)\mathrm{F}\left({\mathrm{s}}_{\mathrm{j}}\right)}\to\ \frac{1}{\upsigma +\mathrm{n}\left({\mathrm{s}}_{\mathrm{j}}\right)}\ \left(\upsigma +\mathrm{n}\left({\mathrm{s}}_{\mathrm{j}}\right)\frac{\mathrm{F}\left({\mathrm{u}}_{\mathrm{i}},{\mathrm{s}}_{\mathrm{j}}\right)}{\mathrm{F}\left({\mathrm{u}}_{\mathrm{i}}\right)\mathrm{F}\left({\mathrm{s}}_{\mathrm{j}}\right)}\right),\hfill \\ {}\hfill \frac{\mathrm{F}\left({\mathrm{u}}_{\mathrm{i}},{\mathrm{s}}_{\mathrm{j}},\mathrm{s}{\hbox{'}}_{\mathrm{k}}\right)}{\mathrm{F}\left({\mathrm{u}}_{\mathrm{i}}\right)\mathrm{F}\left({\mathrm{s}}_{\mathrm{j}},\mathrm{s}{\hbox{'}}_{\mathrm{k}}\right)}\to\ \frac{1}{\upsigma +\mathrm{n}\left({\mathrm{s}}_{\mathrm{j}},\mathrm{s}{\hbox{'}}_{\mathrm{k}}\right)}\ \left(\upsigma +\mathrm{n}\left({\mathrm{s}}_{\mathrm{j}},\mathrm{s}{\hbox{'}}_{\mathrm{k}}\right)\frac{\mathrm{F}\left({\mathrm{u}}_{\mathrm{i}},{\mathrm{s}}_{\mathrm{j}},\mathrm{s}{\hbox{'}}_{\mathrm{k}}\right)}{\mathrm{F}\left({\mathrm{u}}_{\mathrm{i}}\right)\mathrm{F}\left({\mathrm{s}}_{\mathrm{j}},\mathrm{s}{\hbox{'}}_{\mathrm{k}}\right)}\right),\hfill \end{array} $$

with n (s_j_) and n (s_j_,s′_k_) the number of residues of these types in the learning set, and σ = 10. This correction ensures that the “potentials” tend to 0 when the number of observations in the data set is too small.

Using these “potentials”, we computed an energy-like contribution for each residue i in a protein sequence, which measures their propensity of being an epitope (u = E) or a non-epitope (u = N):

4 $$ \begin{array}{cc}\hfill {\Delta \mathrm{W}}_1\left({\mathrm{u}}_{\mathrm{i}}\right)={\displaystyle \sum_j}{\Delta \mathrm{w}}_1\left({\mathrm{u}}_{\mathrm{i}},{\mathrm{s}}_{\mathrm{j}}\right);\hfill & \hfill {\Delta \mathrm{W}}_2\left({\mathrm{u}}_{\mathrm{i}}\right)={\displaystyle \sum_{j, k}}{\Delta \mathrm{w}}_2\left({\mathrm{u}}_{\mathrm{i}},{\mathrm{s}}_{\mathrm{j}},\mathrm{s}{\hbox{'}}_{\mathrm{k}}\right),\hfill \end{array} $$

with j and k in a sequence interval I around residue i. We consider as feature F9 the sum ΔW(u_i_) = ΔW_1_(u_i_) + ΔW_2_(u_i_). The values of this feature, for different amino acids and amino acid pairs, are given in Additional file 2.

#### Solvent accessibility

As epitopes are located at the protein surface, an indispensable feature is the predicted solvent accessibility. We used two different programs for that purpose. The first is NetSurfP [54] (F10), which not only predicts the secondary structure but also classifies residues in buried (B) and exposed residues (E). The second (F11) is an energy-like solvent accessibility predictor that is similar to the epitope/non-epitope predictor described in Eqs (1–4) with the state u_i_ of residue i being exposed (u = E) or buried (u = B). We define a residue to be in the state E (B) if its solvent accessibility is higher (lower) than 5%. This quite low percentage was chosen to ensure that all epitope residues are exposed [29]. The values of this feature, for different amino acids and amino acid pairs, are given in Additional file 3.

#### Solubility

The intrinsic solubility of the amino acid residues in a sequence is closely related to their propensity of having a certain solvent accessibility. The solubility per residue was calculated using the sequence-based version of the CamSol [63] program (F12).

#### Evolutionary information

It is a priori not obvious whether epitope residues are equally conserved during evolution than non-epitope residues. To analyze this, we evaluated the evolutionary conservation of the epitope and non-epitope residues using the position-specific scoring matrix (PSSM) obtained by aligning the target antigen sequence against a non-redundant set of protein sequences with the PSI-BLAST [64] tool. The so obtained conservation value per residue was used as feature F13.

### Feature windows

As the characteristics of a residue are influenced not only by the residue itself but also by the neighboring amino acids along the chain, we considered the features in a sequence window around the targeted residue to predict the epitope/non-epitope state of the latter. Note that this effect is already built in the energy-like features. We considered windows W from 3 to 9-residues centered on the central residue.

### Machine learning

We applied two machine-learning methods to classify residues as epitopes or non-epitopes on the basis of the 13 features F1-F13. These are the Gaussian Naïve Bayes [65] and Random Forest [66] algorithms. All the parameters of these classifiers were optimized first; in particular, better performances were achieved using Random Forest with 100 trees. The predictions of these two basic classifiers were then combined using a voting algorithm, based on averages of the predicted probabilities. This technique tends to balance out the weaknesses of individual machine-learning classifiers. We used the implementation of these techniques in the scikit-learn [67] package.

### Imbalanced dataset

One of the difficulties in predicting epitopes is the strongly imbalanced dataset. Indeed, the number of epitopes is one order of magnitude smaller than the number of non-epitopes. All classifiers that use this dataset for training tend to predict every residue as non-epitope. We tested several standard techniques to deal with such imbalanced data. We finally selected a variant of the SMOTE [68] algorithm, i.e. the SVM SMOTE algorithm [69]. The SMOTE algorithm proceeds by oversampling the minority class by creating synthetic instances using a k-nearest-neighbor approach. Similarly, the SVM SMOTE is an oversampling method that uses a Support Vector Machines (SVM) classifier to create new instances of the minority class. This approach was implemented with the Imbalanced-learn python toolbox [70], which is compatible with scikit-learn. We optimized the parameters of the radial basis function kernel in SVM; the parameters achieving the highest scores were C = 1 and γ = 0.01.

In this way, the size of the original dataset was changed, leading to roughly the same number of epitope and non-epitope residues. This procedure was found to be superior to the common method consisting in dividing the majority class randomly into N equal parts (N being equal to the ratio of non-epitopes to epitopes) and combining each part with the minority class to form N distinct learning sets.

### Performance evaluation metrics

**Fig. 1:**
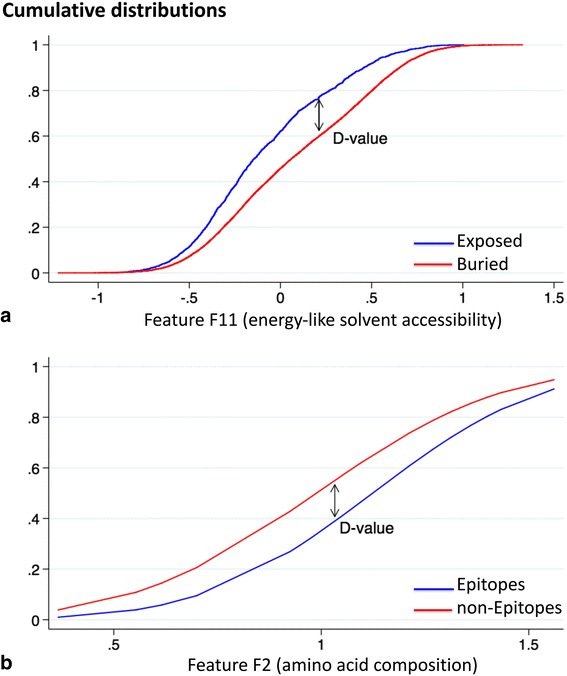
Cumulative distributions for individual features, with the D-value of the KS test indicated (a) Energy-like solvent accessibility feature F11 for the sequence interval of size I = 7, with a D-value of 0.185; (b) Feature F2 defined as the ratio of the amino acid frequency in epitopes and in the remaining antigen, with a D-value of 0.177

The Kolmogorov-Smirnov (KS) statistic [71, 72] is a nonparametric test that quantifies a distance (D-value) between the empirical distribution function of two samples, as shown in Fig. 1. We used it for analyzing, for each feature, the difference – if any - between the distributions observed for epitope and non-epitope residues.

The performance of the prediction models was measured by the area under the receiver operating characteristic (ROC) curve (AUC) [73]. This curve is obtained by plotting the true positive rate (TP) against the false positive rate (FP) for various threshold values, and illustrates the performance of binary classifiers.

The prediction performances were evaluated in 10-fold cross validation on the *S85* set, as well as on the independent test set *S19*. Note that in the 10-fold cross validation procedure, the dataset was split before applying the SVM SMOTE algorithm (see section “Imbalanced dataset” here above), to avoid introducing similarities between the training and test sets. The different classifiers were applied on the same training and test folds.

## Results and discussion

The 14 amino acid-based physicochemical, energy-like, evolutionary and statistical features ﻿F1-F﻿1﻿﻿﻿4﻿ described in Methods were first evaluated separately and then combined to build our B-cell epitope predictor SEPIa. Importantly, all the features were calculated on the basis of the sole amino acid sequences of the antigens and do not require any structural information.

### Feature analysis

We first analyzed the 14 features separately, and compared their capacity to distinguish epitope from non-epitope residues. We used therefore the statistical KS-test, which involves computing the D-value that corresponds to the largest vertical distance between the cumulative distributions of epitope and non-epitope samples computed from the *S85* learning set (See Methods and Fig. 1). Another measure that we used for that purpose is based on the construction of a prediction model using the Gaussian Naïve Bayes and Random Forest machine learning algorithms, and combining them using a voting procedure, as explained in Methods. The AUC score of this prediction model, calculated in 10-fold cross validation on the *S85* set, yields another estimation of the informative power of the individual features.

A novel sequence-based characteristic introduced in this study in the context of B-cell epitope prediction consists of the energy-like features ΔW_1_ (E/N) and ΔW_2_ (E/N) (Eq. (4)), which compute the influence of single amino acids and amino acid pairs, respectively, in a sequence interval of I residues centered around a central residue on the epitope/non-epitope state on this central residue. To identify the optimal size of I, we first used the KS-test. For all tested sizes, ranging for I = 3 to 17, the cumulative distributions of energy values for epitope and non-epitope residues were found to be significantly different (*P*-value < 0.0001). The best size, determined as the one that gives the largest D-value, is I = 3 and I = 7 almost ex-aequo for ΔW_1_, and I = 7 for ΔW_2_. Testing the informative value of these features on the basis of the AUC of the prediction model also selects I = 7 as the optimal sequence interval size. We hence fixed I = 7, and considered as feature F9 the sum of the two potentials, ΔW (E/N) = ΔW_1_ (E/N) + ΔW_2_ (E/N). The cumulative distributions for E/N states obtained from this feature are slightly better separated than for the individual potentials, with a D-value of 0.166 and an AUC score of 0.551 (Table 1).

**Table 1:** Prediction performance of the individual features F1-13 and of their combination (F), for all window sizes W = 0-9, estimated by the AUC score and evaluated by 10-fold cross validation of the *S85* set. The features indicate intrinsically disordered regions (F8 and F7), flexibility (F5 and F6), evolutionary information (F13), energy-like (F9), secondary structure (F4), solvent accessibility (F10 and F11), solubility (F12), hydrophilicity (F3), and amino acid composition (F1 and F2)

	AUC score for different window sizes W													
W	F1	F2	F3	F4	F5	F6	F7	F8	F9	F10	F11	F12	F13	F
0	0.586	0.574	0.545	0.561	0.517	0.560	0.523	0.519	0.551	0.516	0.521	0.547	0.532	0.644
3	0.591	0.615	0.576	0.533	**0.544**	0.579	0.543	0.514	0.569	0.548	0.542	0.585	0.547	0.639
5	0.604	0.597	0.579	0.552	0.542	**0.580**	0.544	0.511	**0.580**	0.583	0.575	0.588	**0.554**	0.635
7	0.600	0.603	0.570	0.558	0.541	**0.580**	0.545	0.495	**0.580**	**0.590**	**0.609**	**0.591**	0.548	0.640
9	**0.614**	**0.619**	**0.593**	**0.560**	0.533	0.579	**0.557**	**0.525**	0.553	0.569	0.586	0.570	0.550	**0.646**

Values in bold correspond to the optimal window sizes for each feature

Epitope residues are always located near the surface, and solvent accessibility is thus obviously an important epitope characteristic. We introduced an energy-like feature that compiles solvent accessibility propensities in much the same way than the energy-like epitope/non-epitope feature analyzed above. In this case the state of a residue is surface/buried (S/B) rather than epitope/non-epitope. The optimal sequence interval size I was evaluated on the basis of the KS D-value and the AUC score of the prediction model. Again, I = 7 appears as the best compromise, both for the feature ΔW_1_ (S/B) based on individual amino acid propensities and ΔW_2_ (S/B) based on pairs of amino acids. The sum of these two potentials, ΔW (S/B) = ΔW_1_ (S/B) + ΔW_2_ (S/B) with I = 7 is defined as feature F11, and leads to cumulative distributions that are slightly better separated than the individual ones, with a D-value of 0.185 (Fig. 1), and an AUC score of 0.521 (Table 1).

Note that the other energy-like feature F11, which is based on the preference of amino acids to be at the surface or buried, distinguishes better epitopes from non-epitopes than feature F9, which is based on the preference of amino acids to be (non-) epitopes. This apparent discrepancy is due to the fact that the epitope/non-epitope assignments in the *S85* dataset include more errors than the surface/buried assignments. Indeed, the latter are obtained from the structure and thus are basically error-free. The epitope residues are also correctly assigned, as they are obtained from the structures of the antigen/antibody complexes. In contrast, some of the residues assigned as non-epitopes are probably epitopes in other antigen/antibody complexes. This obviously induces noise in the epitope learning dataset.

Besides the features F9 and F11, we tested the informative content of all other features F1-F14. According to the KS-test, the features F1-F13 differ significantly between the epitope and non-epitope samples with *P*-values < 0.05, and have higher than random AUC scores (see Table 1). Only the β-turn feature F14 did not show a statistically significant difference between epitope and non-epitope residues. Indeed, the KS-test D-value was found to be equal to 0.028 with a P-value of 0.183, and the AUC score is 0.506. We thus dropped this feature and kept the 13 features F1-F13 for building the epitope predictor.

The characteristics of a residue are influenced not only by the residue itself but also by the neighboring residues along the chain. They are also influenced by the residues that are in spatial contact, but as we restrict ourselves to sequence-based features, we cannot consider them. We tested the information gain obtained with sequence windows W between 3 to 9 residues. Note that these windows are related to the sequence intervals considered for the two energy-like features F9 and F11, for which the optimal value was I = 7. These intervals are considered in the feature construction, whereas the windows are used at the level of the prediction. We tested varying window sizes W in designing the predictor for all features including F9 and F11.

The results of the AUC scores, computed in 10-fold cross validation, are given in Table 1 for the different window sizes and the 13 individual features. For 7 out of the 13 features, the optimal value is W = 9. We thus selected this window size.

**Fig. 2:**
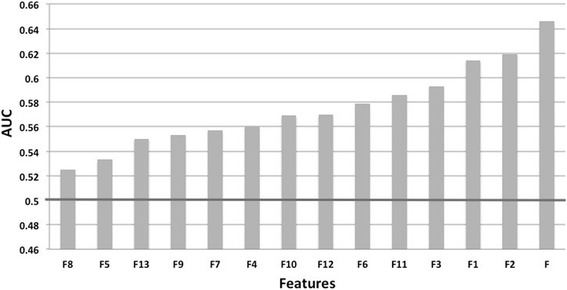
Prediction performance of the individual features F1-13 and of their combination (F), estimated by the AUC and evaluated by 10-fold cross validation of the *S85* set, using a sequence window size W = 9. The bold horizontal line indicates the level of random prediction. From least to best performing: intrinsically disordered regions (F8 and F7), flexibility (F5 and F6), evolutionary information (F13), energy-like (F9), secondary structure (F4), solvent accessibility (F10 and F11), solubility (F12), hydrophilicity (F3), and amino acid composition (F1 and F2)

The 13 features are ranked as a function of increasing AUC in Fig. 2 (for W = 9). The best individual features are F1, F2 and F3, and are related to the amino acid composition. The best one, F2, is equal to the ratio of amino acid frequencies in epitopes and in the remaining antigen, and reaches an AUC of 0.62. The second best, F1, is the ratio of the amino acid frequency in epitopes and in the remaining antigen surface, and has an AUC of 0.61. The third best feature, F3, is hydrophilicity, with an AUC score of 0.59. The energy-like epitope/non-epitope feature F9, which is based on more complex combinations of amino acid propensities, is slightly less performing, with an AUC of 0.55. Note that it is higher (0.58) for W = 5 and 7.

The next best features are related to the solvent accessibility prediction, which is obviously an important factor since epitopes are at the protein surface. The best of these is the energy-like accessibility feature F11, with an AUC of 0.59, followed by the solvent accessibility feature F10, based on NetSurfP predictions, with an AUC of 0.57. The related feature F12, solubility, also presents an AUC of 0.57.

The flexibility features F5 and F6 also carry some information. F6, obtained from the RMSF computed from molecular dynamics simulations, shows an AUC of 0.58, while F5, obtained from backbone N-H S^2^ order parameters, is only slightly above random, with an AUC of 0.53. This reflects the observations that epitope residues are usually positioned in flexible regions. Similarly, epitopes are more often localized in loop regions than in helices and strands; the predicted secondary structure, feature F4, has an intermediate AUC of 0.56. The related features F7 and F8, based on the prediction of intrinsically disordered regions, have AUC values in the same range: 0.56 and 0.53, respectively.

The last feature, F13, which is based on evolutionary sequence conservation, has a low AUC of 0.55: epitope regions are slightly less conserved than other regions, but the difference is small.

Overall, the analysis of the individual features indicates that all the tested attributes possess a weak to medium ability of differentiating epitope from non-epitope residues.

### SEPIa predictor

We combined the 13 tested features to set up the final predictor, called SEPIa. The algorithm used is the same as for the individual features, a combination of Gaussian Naïve Bayes and Random Forest algorithms using a voting procedure (see Methods). Note that even those features that are only slightly better than random carry some information. Indeed, their elimination decreases the prediction score.

The results obtained in 10-fold cross validation procedure on the *S85* set are given in Table 1 for window sizes W = 0-9 (column F). The best AUC scores are obtained for W = 9, like for the individual features. They reach 0.65, which is small but significant increase with respect to the individual features. Indeed, the best feature, F2, reached only 0.62. The gradual improvement of the overall performance upon sequential addition of the features is given in Table 2.

**Table 2:** Increase of the prediction performance upon sequential addition of features. The window size is W = 9, and the AUC score is evaluated in 10-fold cross validation on the *S85* dataset

Feature combination	AUC score
F1	0.619
F1 + F2	0.624
F1 + F2 + F10	0.629
F1 + F2 + F10 + F11	0.630
F1 + F2 + F10 + F11 + F12	0.631
F1 + F2 + F9 + F10 + F11 + F12	0.631
F1 + F2 + F6 + F9 + F10 + F11 + F12	0.636
F1 + F2 + F3 + F6 + F9 + F10 + F11 + F12	0.636
F1 + F2 + F3 + F6 + F9 + F10 + F11 + F12 + F13	0.637
F1 + F2 + F3 + F6 + F9 + F10 + F11 + F12 + F13 + F7	0.640
F1 + F2 + F3 + F6 + F9 + F10 + F11 + F12 + F13 + F7 + F4	0.644
F1 + F2 + F3 + F6 + F9 + F10 + F11 + F12 + F13 + F7 + F4 + F5	0.644
F1 + F2 + F3 + F6 + F9 + F10 + F11 + F12 + F13 + F7 + F4 + F5 + F8	**0.646**

The largest AUC score is indicated in bold

Two of the antigens of the *S85* training set have residues that are epitopes in some antigen-antibody complexes and non-epitopes in others. As most such common epitopes have been found related to autoimmunity [74], we removed these antigens from the *S85* set, and trained another model on this restricted set *S83* (Additional file 1: Table S1). The AUC obtained in 10-fold cross validation is equal to 0.65, and is thus identical to that obtained from the full dataset. We thus chose to keep the SEPIa predictor obtained with the complete *S85* training set.

We also tested the SEPIa predictor on *S19*, an independent dataset of 19 antigen sequences [42], whose epitope assignment was made on the basis of experimental (non-structural) data (see Methods), and which is here used for comparison with other methods (see next section). The results on this test set were quite similar to those obtained from *S85*, as shown in Table 3: the window size 9 appears to be the best, and the AUC score reaches 0.65. The agreement between the results obtained from these two independent datasets increases their confidence level.

**Table 3:** Prediction performance of the combination of features as a function of the window size, estimated by the AUC score and evaluated on the *S19* test set

Window size	AUC score
0	0.643
3	0.639
5	0.635
7	0.640
9	**0.646**

The best score is indicated in bold

The SEPIa prediction model, obtained with the scikit-learn package, is available as a file SEPIa.zip in Additional file 4. It needs as input the sequence of the target protein, and the 13 features computed on it.

### Comparison with other methods

Several other B-cell epitope prediction methods have been developed, of which a certain number have been tested on the *S19* set, while trained on an independent set. These are: Zhang_bound_ and Zhang_unbound_ [45], Zheng_bound_ and Zheng_unbound_ [52], CBTOPE [75], EPCES [76], Epitopia [41], DiscoTope [34], BPredictor [43], SEPPA [39], and EPSVR [42]. The former five use the amino acid sequence as sole input, whereas the last six also use the 3D structure. The AUC scores obtained by these methods are given in Table 4; they are taken from the original articles and from [45].

**Table 4:** The performance of different epitope prediction servers, estimated by the AUC score and evaluated on the *S19* test set

Category	Method	AUC
Sequence- based	Ensemble_bound_ [52]	0.579
	Zhang_bound_ [45]	0.600
	Zhang_unbound_ [45]	0.601
	Ensemble_unbound_ [52]	0.604
	CBTOPE [74]	0.607
	SEPIa	**0.646**
Structure-based	EPCES [75]	0.569
	EPITOPIA [41]	0.572
	DiscoTope [34]	0.579
	BPredictor [43]	0.587
	SEPPA [39]	0.589
	EPSVR [42]	0.606

The largest score is indicated in bold

With its AUC score of 0.65, SEPIa appears to slightly outperform the other methods. The second best sequence-based method, CBTOPE, has an AUC of 0.61. Note, however, that the *S19* dataset is too small for these score differences to be statistically significant.

The structure-based methods do not perform better than the sequence-based methods. The best one, EPSVR, has an AUC of 0.61. This can seem surprising, given that considering the 3D structure obviously adds information. However, in this case, the predictors focus on surface residues only and classify them into epitope and non-epitope regions. Therefore, the scores of the sequence- and structure-based predictors cannot be compared: the former distinguish epitope residues out of all surface and core residues, and the latter epitope residues out of surface residues only.

### Case study

**Fig. 3:**
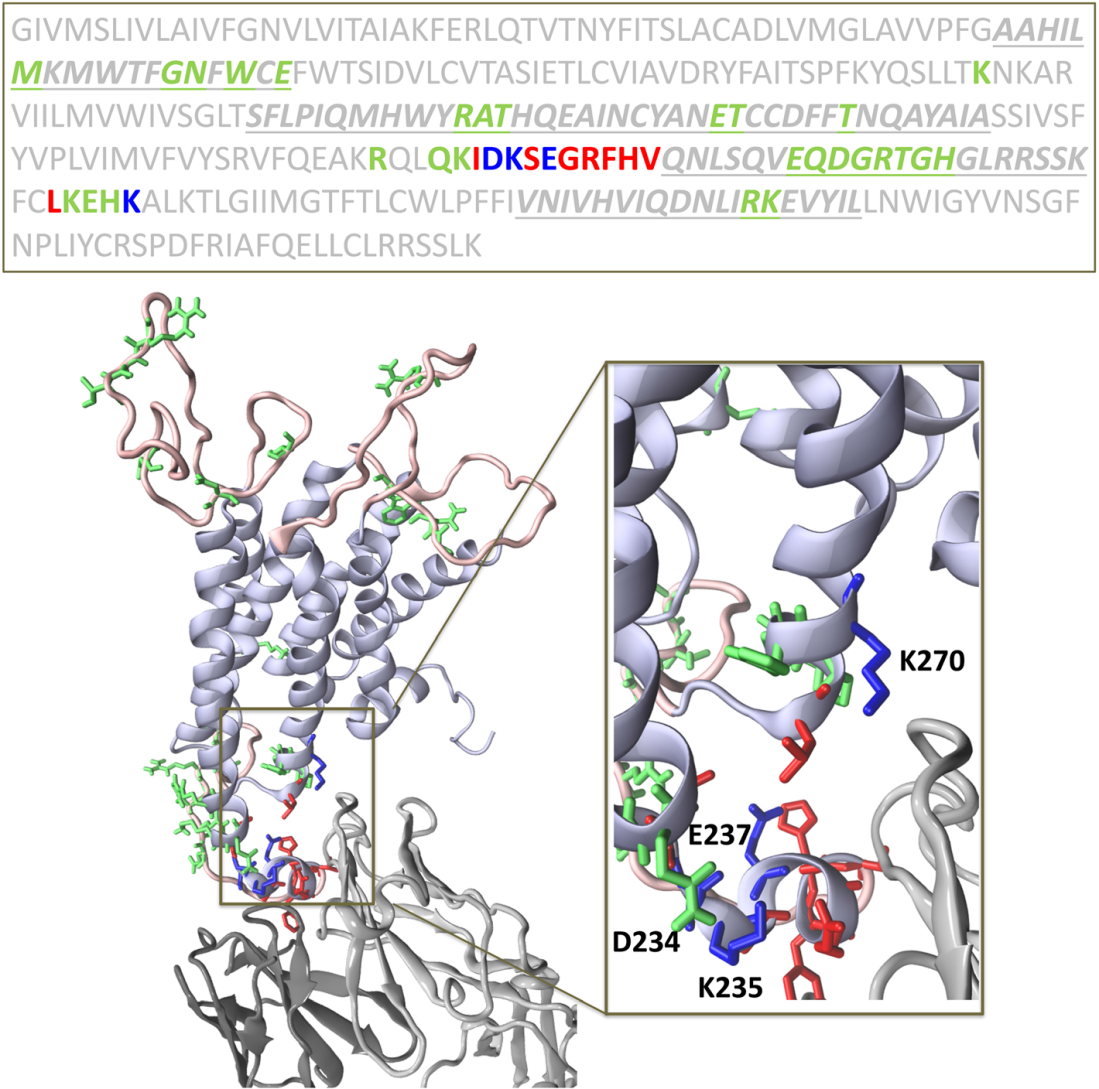
Predicted and observed epitope residues in the human β2AR receptor. The predicted epitope residues are in green, the observed epitopes are in red, and the residues that are both predicted and observed as epitopes are in blue. Above: amino acid sequence, with the modeled loop regions in italic and underlined. Below: structure of β2AR co-crystallized with a Fab fragment, shown as ribbons with predicted and observed epitopes in sticks; β2AR is colored in light purple with modeled regions in light pink, Fab heavy chain in dark gray and Fab light chain in light gray

To test the performance of the SEPIa predictor, we applied it on the human β2 adrenergic G-protein-coupled receptor (β2AR), which was co-crystallized with an antigen-binding fragment (Fab) and solved by X-ray crystallography (PDB ID: 2R4R) [77]. Due to its low resolution of 3.4 Å, it was excluded from the learning set *S85*. Its sequence identity with the antigens from the *S85* learning set is below 20%, which makes it a good case study. Several sequence regions are absent in the X-ray structure: the C-terminus, the three extracellular loops and the third intracellular loop. Comparative modeling with the help of the SwissModel [78] server was used to build a complete structural model of β2AR, using the 2R4R structure as a template. The structure of the complex is depicted in Fig. 3.

The epitope residues were assigned from the structure of the antibody-antigen complex as described in Methods. There are 12 epitope residues, depicted in red and blue in Fig. 3. The predictions obtained with SEPIa involve 29 epitope residues (in green and blue). Only 4 epitopes are both predicted and observed (blue). Hence, the number of correctly predicted epitopes is TP = 4, of correctly predicted negatives TN = 272, of incorrectly predicted epitopes FP = 8, and of incorrectly predicted non-epitopes FN = 28. The AUC score on this test protein is equal to 0.77.

Although the score is quite low, it is worth looking in more detail at the predictions shown in Fig. 3. Six residues that are incorrectly predicted as epitopes are actually very close to the binding interface. Adding the 4 correctly predicted epitopes yields a total of 10 residues that overlap the actual epitope region of 12 residues. The 18 other incorrectly predicted epitopes are all but one situated in loop regions at the protein surface; the fact that SEPIa does not predict epitopes in the core – on the basis of the sole amino acid sequence - is in itself already a positive result. Moreover, the other incorrectly predicted epitopes do not form clusters of interacting residues, except in one region. Note that some of these incorrectly predicted epitopes could very well correspond to epitopes in another complex, with another antibody, as discussed above.

## Conclusions

The identification of immunogenic regions on antigen proteins provides the basis for the rational design of potential vaccines. In this study, we have developed the SEPIa conformational epitope predictor, which is based on the amino sequence alone. It uses a voting algorithm for combining the predictions of two classifiers, a Gaussian Naïve Bayes and a Random Forest classifier. This procedure tends to alleviate the weaknesses of the individual models. Thirteen amino acid-based features were exploited in the predictor. Some of them are directly linked to the amino acid sequence and describe the frequency, physicochemical properties, and evolutionary conservation of the amino acid residues. The others are sequence-based predictions of different features, in particular the epitope state, solvent accessibility, secondary structure, flexibility, and disorder. The values of all these features in a sequence window of 9 residues are taken into account to predict whether or not the middle residue is an epitope. Our method achieves an AUC score of 0.65 in 10-fold cross validation on the learning dataset. Almost the same performance is obtained on an independent dataset, on which other predictors have also been tested.

Strikingly, the performance of the SEPIa predictor, albeit limited, outperforms other methods. Moreover, its application to a test protein, β2AR, demonstrated its usefulness. First, many of the predicted epitope residues in this protein are either correctly predicted or spatially close to the experimentally determined epitope residues. Furthermore, most incorrectly predicted epitope residues are located in loops – and could thus be assumed to correspond to different, not yet identified, epitopes -, and/or do not cluster together in space – which could allow to mark them as unlikely epitopes. This last characteristic opens new perspectives for proteins of known or modeled structure, which involves combining the predicted epitope residues that are close in space into epitope clusters, with the largest clusters being more likely to correspond to true epitope regions.

Finally, we would like to underline two difficulties which all epitope predictors are faced with, and which limit their performances. The first is the noisy learning dataset, due to the fact that some residues marked as non-epitopes are in fact epitopes in other antigen-antibody complexes. The second is the strong imbalance between the number of epitope and non-epitope residues, which impedes high-performance machine learning. The last is related to the observation that proteins can exist and be stable without being recognized by antibodies. This implies that the properties of epitope residues are not very different from those of other surface residues, and increases the complexity of the prediction issue.

## Additional files

Additional file 1: List of antigen proteins of the *S85* and *S83* datasets (Table S1) and of the *S19* dataset (Table S2), and values of the amino acid frequency-based features F1 and F2 (Table S3). (DOCX 34 kb)
Additional file 2: Values of the epitope/non-epitope energy-like feature F9. (ZIP 61 kb)
Additional file 3: Values of the solvent accessibility energy-like feature F11. (ZIP 129 kb)
Additional file 4: SEPIa prediction model, implemented using the scikit-learn package. (ZIP 3901 kb)

## Abbreviations

3D: Three-dimensional
AUC: Area under the ROC curve
CDR: Complementarity determining region
CED: Conformational epitope database
Fab: Fragment antigen-binding
FP: False positive rate
IEDB-3D: Immune Epitope Database
KS: Kolmogorov-Smirnov
PDB: Protein Data Bank
PSSM: Position-specific scoring matrix
RMSF: Root mean square fluctuations
ROC: Receiver operating characteristic
TP: True positive
β2AR: Human β2 adrenergic G-protein-coupled receptor

## References

[1] Irving MB, Pan O, Scott JK (2001). Random-peptide libraries and antigen-fragment libraries for epitope mapping and the development of vaccines and diagnostics. Curr Opin Chem Biol.

[2] Regenmortel MHV Van: Epitope Mapping Protocols. 2009, 524. [Methods in Molecular Biology^TM^] 19514158

[3] Gershoni JM, Roitburd-Berman A, Siman-Tov DD, Tarnovitski Freund N, Weiss Y (2007). Epitope Mapping. BioDrugs.

[4] Barlow DJ, Edwards MS, Thornton JM (1986). Continuous and discontinuous protein antigenic determinants. Nature.

[5] El-Manzalawy Y, Honavar V (2010). Recent advances in B-cell epitope prediction methods. Immunome Res.

[6] Hopp TP, Woods KR (1981). Prediction of protein antigenic determinants from amino acid sequences. Proc Natl Acad Sci U S A.

[7] Parker JM, Guo D, Hodges RS (1986). New hydrophilicity scale derived from high-performance liquid chromatography peptide retention data: correlation of predicted surface residues with antigenicity and X-ray-derived accessible sites. Biochemistry.

[8] Pellequer JL, Westhof E, Van Regenmortel MHV. Predicting location of continuous epitopes in proteins from their primary structures. In: Langone JJ, editor, Methods of Enzymology. San Diego: Academic Press; 1991;203:176–201. 10.1016/0076-6879(91)03010-e 1722270

[9] Emini EA, Hughes JV, Perlow DS, Boger J (1985). Induction of hepatitis A virus-neutralizing antibody by a virus-specific synthetic peptide. J Virol.

[10] Karplus PA, Schulz GE (1985). Prediction of chain flexibility in proteins. Naturwissenschaften.

[11] Kolaskar AS, Tongaonkar PC (1990). A semi-empirical method for prediction of antigenic determinants on protein antigens. FEBS Lett.

[12] Welling GW, Weijer WJ, van der Zee R, Welling-Wester S (1985). Prediction of sequential antigenic regions in proteins. FEBS Lett.

[13] Blythe MJ, Flower DR (2005). Benchmarking B cell epitope prediction: Underperformance of existing methods. Protein Sci.

[14] Larsen JEP, Lund O, Nielsen M (2006). Improved method for predicting linear B-cell epitopes. Immunome Res.

[15] Saha S, Raghava GPS: Prediction of Continuous B-Cell Epitopes in an Antigen Using Recurrent Neural Network. Bioinformatics 2006, 48(May 2005):40–48 10.1002/prot.21078 16894596

[16] Chen J, Liu H, Yang J, Chou K-C (2007). Prediction of linear B-cell epitopes using amino acid pair antigenicity scale. Amino Acids.

[17] El-Manzalawy Y, Dobbs D, Honavar V (2008). Predicting linear B-cell epitopes using string kernels. J Mol Recognit JMR.

[18] Wee LJ, Simarmata D, Kam Y-W, Ng LF, Tong JC (2010). SVM-based prediction of linear B-cell epitopes using Bayes Feature Extraction. BMC Genomics.

[19] Wang Y, Wu W, Negre NN, White KP, Li C, Shah PK (2011). Determinants of antigenicity and specificity in immune response for protein sequences. BMC Bioinformatics.

[20] Gao J, Faraggi E, Zhou Y, Ruan J, Kurgan L (2012). BEST: improved prediction of B-cell epitopes from antigen sequences. PLoS One.

[21] Yao B, Zhang L, Liang S, Zhang C (2012). SVMTriP: A Method to Predict Antigenic Epitopes Using Support Vector Machine to Integrate Tri-Peptide Similarity and Propensity. PLoS One.

[22] Sweredoski MJ, Baldi P (2009). COBEpro: a novel system for predicting continuous B-cell epitopes. Protein Eng Des Sel.

[23] Lian Y, Ge M, Pan X-M (2014). EPMLR: sequence-based linear B-cell epitope prediction method using multiple linear regression. BMC Bioinformatics.

[24] Van Regenmortel MH (1996). Mapping Epitope Structure and Activity: From One-Dimensional Prediction to Four-Dimensional Description of Antigenic Specificity. Methods.

[25] Rubinstein ND, Mayrose I, Halperin D, Yekutieli D, Gershoni JM, Pupko T (2008). Computational characterization of B-cell epitopes. Mol Immunol.

[26] Ofran Y, Schlessinger A, Rost B (2008). Automated identification of complementarity determining regions (CDRs) reveals peculiar characteristics of CDRs and B cell epitopes. J Immunol.

[27] Sun J, Xu T, Wang S, Li G, Wu D, Cao Z (2011). Does difference exist between epitope and non-epitope residues? Analysis of the physicochemical and structural properties on conformational epitopes from B-cell protein antigens. Immunome Res.

[28] Kringelum JV, Nielsen M, Padkjær SB, Lund O (2013). Structural analysis of B-cell epitopes in antibody:protein complexes. Mol Immunol.

[29] Dalkas GA, Teheux F, Kwasigroch JM, Rooman M (2014). Cation-π, amino-π, π-π, and H-bond interactions stabilize antigen-antibody interfaces. Proteins Struct Funct Bioinforma.

[30] Jones S, Thornton JM (1997). Prediction of protein-protein interaction sites using patch analysis. J Mol Biol.

[31] Jones S, Thornton JM (1997). Analysis of protein-protein interaction sites using surface patches. J Mol Biol.

[32] Jones S, Thornton J (1996). Principles of protein-protein interactions. Proc Natl Acad Sci.

[33] Lo Conte L, Chothia C, Janin J (1999). The atomic structure of protein-protein recognition sites. J Mol Biol.

[34] Andersen PH, Nielsen M, Lund O (2006). Prediction of residues in discontinuous B‐cell epitopes using protein 3D structures. Protein Sci.

[35] Sweredoski MJ, Baldi P (2008). PEPITO: improved discontinuous B-cell epitope prediction using multiple distance thresholds and half sphere exposure. Bioinformatics.

[36] Ponomarenko J, Bui H-H, Li W, Fusseder N, Bourne PE, Sette A, Peters B (2008). ElliPro: a new structure-based tool for the prediction of antibody epitopes. BMC Bioinformatics.

[37] Rapberger R, Lukas A, Mayer B (2007). Identification of discontinuous antigenic determinants on proteins based on shape complementarities. J Mol Recognit..

[38] Ren J, Liu Q, Ellis J, Li J (2014). Tertiary structure-based prediction of conformational B-cell epitopes through B factors. Bioinformatics.

[39] Sun J, Wu D, Xu T, Wang X, Xu X, Tao L, Li YX, Cao ZW. SEPPA: a computational server for spatial epitope prediction of protein antigens. Nucleic Acids Res. 2009;37(2):W612-6. 10.1093/nar/gkp417 PMC2703964 19465377

[40] Rubinstein ND, Mayrose I, Pupko T (2009). A machine-learning approach for predicting B-cell epitopes. Mol Immunol.

[41] Rubinstein ND, Mayrose I, Martz E, Pupko T (2009). Epitopia: a web-server for predicting B-cell epitopes. BMC Bioinformatics.

[42] Liang S, Zheng D, Standley DM, Yao B, Zacharias M, Zhang C (2010). EPSVR and EPMeta: prediction of antigenic epitopes using support vector regression and multiple server results. BMC Bioinformatics.

[43] Zhang W, Xiong Y, Zhao M, Zou H, Ye X, Liu J (2011). Prediction of conformational B-cell epitopes from 3D structures by random forest with a distance-based feature. BMC Bioinformatics.

[44] Hu Y-J, Lin S-C, Lin Y-L, Lin K-H, You S-N (2014). A meta-learning approach for B-cell conformational epitope prediction. BMC Bioinformatics.

[45] Zhang W, Niu Y, Xiong Y, Zhao M, Yu R, Liu J (2012). Computational prediction of conformational B-cell epitopes from antigen primary structures by ensemble learning. PLoS One.

[46] Habibi M, Bakhshi PK, Aghdam R (2015). LRC: A new algorithm for prediction of conformational B-cell epitopes using statistical approach and clustering method. J Immunol Methods.

[47] Ren J, Liu Q, Ellis J, Li J (2015). Positive-unlabeled learning for the prediction of conformational B-cell epitopes. BMC Bioinformatics.

[48] Kittler J, Hatef M (1998). On combining classifiers. IEEE Trans Pattern Anal Mach Intell.

[49] Ponomarenko J, Papangelopoulos N, Zajonc DM, Peters B, Sette A, Bourne PE (2011). IEDB-3D: structural data within the immune epitope database. Nucleic Acids Res.

[50] Thompson JD, Higgins DG, Gibson TJ (1994). CLUSTAL W: improving the sensitivity of progressive multiple sequence alignment through sequence weighting, position-specific gap penalties and weight matrix choice. Nucleic Acids Res.

[51] Berman HM, Westbrook J, Feng Z, Gilliland G, Bhat TN, Weissig H, Shindyalov IN, Bourne PE (2000). The Protein Data Bank. Nucleic Acids Res.

[52] Zheng W, Zhang C, Hanlon M, Ruan J, Gao J (2014). An ensemble method for prediction of conformational B-cell epitopes from antigen sequences. Comput Biol Chem.

[53] Huang J, Honda W (2006). CED: a conformational epitope database. BMC Immunol.

[54] Petersen B, Petersen TN, Andersen P, Nielsen M, Lundegaard C (2009). A generic method for assignment of reliability scores applied to solvent accessibility predictions. BMC Struct Biol.

[55] Singh H, Singh S, Raghava GPS (2015). In silico platform for predicting and initiating β-turns in a protein at desired locations. Proteins.

[56] Cilia E, Pancsa R, Tompa P, Lenaerts T, Vranken WF: The DynaMine webserver: predicting protein dynamics from sequence. Nucleic Acids Res 2014:gku270-. 10.1093/nar/gku270 PMC4086073 24728994

[57] de Brevern AG, Bornot A, Craveur P, Etchebest C, Gelly J-C (2012). PredyFlexy: flexibility and local structure prediction from sequence. Nucleic Acids Res.

[58] Dosztányi Z, Csizmok V, Tompa P, Simon I (2005). IUPred: web server for the prediction of intrinsically unstructured regions of proteins based on estimated energy content. Bioinformatics.

[59] Dosztányi Z, Mészáros B, Simon I (2009). ANCHOR: web server for predicting protein binding regions in disordered proteins. Bioinformatics.

[60] Dehouck Y, Gilis D, Rooman M (2006). A new generation of statistical potentials for proteins. Biophys J.

[61] Sippl MJ (1990). Calculation of conformational ensembles from potentials of mean force. An approach to the knowledge-based prediction of local structures in globular proteins. J Mol Biol.

[62] Rooman MJ, Kocher JP, Wodak SJ (1991). Prediction of protein backbone conformation based on seven structure assignments. Influence of local interactions. J Mol Biol.

[63] Sormanni P, Aprile FA, Vendruscolo M (2015). The CamSol method of rational design of protein mutants with enhanced solubility. J Mol Biol.

[64] Altschul S (1997). Gapped BLAST and PSI-BLAST: a new generation of protein database search programs. Nucleic Acids Res.

[65] Kuncheva LI (2006). On the optimality of Naïve Bayes with dependent binary features. Pattern Recognit Lett.

[66] Breiman L (2001). Random Forests. Mach Learn.

[67] Pedregosa F, Varoquaux G, Gramfort A, Michel V, Thirion B, Grisel O, Blondel M, Prettenhofer P, Weiss R, Dubourg V, Vanderplas J, Passos A, Cournapeau D, Brucher M, Perrot M, Duchesnay E (2011). Scikit-learn: Machine Learning in Python. J Mach Learn Res.

[68] Chawla NV, Bowyer KW, Hall LO, Kegelmeyer WP (2002). SMOTE: Synthetic Minority Over-sampling Technique. J Artif Intell Res..

[69] Nguyen HM, Cooper EW, Kamei K (2011). Borderline over-sampling for imbalanced data classification. Int J Knowl Eng Soft Data Paradig.

[70] Lemaitre G, Nogueira F, Aridas CK: Imbalanced-learn: A Python Toolbox to Tackle the Curse of Imbalanced Datasets in Machine Learning. CoRR 2016, abs/1609.0.

[71] Smirnov NV (1933). Estimate of deviation between empirical distribution functions in two independent samples. Bull Moscow Univ.

[72] Kolmogorov AN (1933). Sulla determinazione empirica di una legge di distribuzione. G dell’ Ist Ital degli Attuari.

[73] Swets JA (1979). ROC analysis applied to the evaluation of medical imaging techniques. Invest Radiol..

[74] Singh H, Ansari HR, Raghava GPS (2013). Improved method for linear B-cell epitope prediction using antigen’s primary sequence. PLoS One.

[75] Ansari HR, Raghava GP (2010). Identification of conformational B-cell Epitopes in an antigen from its primary sequence. Immunome Res.

[76] Liang S, Zheng D, Zhang C, Zacharias M (2009). Prediction of antigenic epitopes on protein surfaces by consensus scoring. BMC Bioinformatics.

[77] Rasmussen SGF, Choi H-J, Rosenbaum DM, Kobilka TS, Thian FS, Edwards PC, Burghammer M, Ratnala VRP, Sanishvili R, Fischetti RF, Schertler GFX, Weis WI, Kobilka BK (2007). Crystal structure of the human beta2 adrenergic G-protein-coupled receptor. Nature.

[78] Biasini M, Bienert S, Waterhouse A, Arnold K, Studer G, Schmidt T, Kiefer F, Gallo Cassarino T, Bertoni M, Bordoli L, Schwede T (2014). SWISS-MODEL: modelling protein tertiary and quaternary structure using evolutionary information. Nucleic Acids Res.

