# Supplementary material for: SEPIa, a knowledge-driven algorithm for predicting conformational B-cell epitopes from the amino acid sequence
Source: BMC Bioinformatics. 2017 Feb 10;18:95. doi: 10.1186/s12859-017-1528-9 (PMC5301386; doi:10.1186/s12859-017-1528-9)
Supplement: Additional file 1: — List of antigen proteins of the S85 and S83 datasets (Table S1) and of the S19 dataset (Table S2), and values of the amino acid frequency-based features F1 and F2 (Table S3). (DOCX 34 kb) [file 12859_2017_1528_MOESM1_ESM.docx]

**Additional File 1 - Supplementary Tables**

| 1A2Y:C | 1IQD:C | 1OSP:O | 2AEP:A | 2J4W:D | 2VXQ:A | 3GJF:A | 3O2D:A |
| --- | --- | --- | --- | --- | --- | --- | --- |
| 1BGX:T | 1JPS:T | 1OTS:A | 2ARJ:R | 2JEL:P | 2VXT:I | 3GRW:A | 3PGF:A |
| 1BJ1:V,W | 1JRH:I | 1PG7:H,L | 2B2X:A | 2NR6:A | 2XQY:E | 3H42:A,B | 3QWO:P |
| 1DVF:A,B | 1KB5:A | 1PKQ:E | 2BDN:A | 2OSL:Q | 2XWT:C | 3HB3:B |  |
| 1E6J:P | 1LK3:A | 1TPX:A | 2CMR:A | 2OZ4:A | 2YC1:C | 3HI6:A |  |
| 1EGJ:A | 1N8Z:C | 1V7M:V | 2DD8:S | 2Q8B:A | 2ZCH:P | 3KR3:D |  |
| 1FE8:A | 1NCA:N | 1WEJ:F | 2FD6:U | 2QQK:A | 3BN9:B | 3L5X:A |  |
| 1FNS:A | 1NFD:B | 1XIW:A | 2H9G:S | 2QQN:A | 3CX5:E | 3L95:Y |  |
| 1FSK:A | 1NL0:G | 1YJD:C | 2HFG:R | 2R0L:A | 3D85:C | 3LEV:A |  |
| 1H0D:C | 1OB1:C | 1YY9:A | 2I5Y:G | 2R56:A | 3GBN:A,B | 3MXW:A |  |
| 1HYS:B | 1ORS:C | 1ZTX:E | 2IH3:C | 2UZI:R | 3GI9:C | 3NIG:A |  |

**Table S1**. The protein antigen chains that constitute the *S85* training dataset. Note that the antigens 1LYS, 1DJQ and 1P2C have a high sequence similarity with 1A2Y, but are in complex with different antibodies and have different epitopes. We kept only one structure, 1A2Y, in which we marked as epitopes all the residues assigned as epitopes in one of the four structures. We did the same with the antigens 1FE8 and 2ADF: we dropped the latter but kept its epitopes. The two antigen chains that are underlined possess common epitopes. The *S83* dataset contains all the antigen chains except these two.

| 1A7C:A | 1EKU:A | 1NU6:A | 1QGT:B | 2B5I:C |
| --- | --- | --- | --- | --- |
| 1AL2:1 | 1JEQ:A | 1OG5:A | 1REC:A | 2GIB:A |
| 1AV1:A | 1LY2:A | 1P4T:A | 1W7B:A | 2GMF:A |
| 1DAB:A | 1MBN:A | 1PV6:A | 1Y8O:B |  |

**Table S2**. The protein antigen chains that constitute the *S19* test dataset.^41^

| Amino acid | Feature F1 | Feature F2 |
| --- | --- | --- |
| W | 1.2344 | 1.0960 |
| Y | 1.3511 | 1.2698 |
| H | 1.2063 | 1.4022 |
| K | 1.2056 | 1.5608 |
| R | 1.1327 | 1.3991 |
| D | 1.2141 | 1.4334 |
| E | 1.0320 | 1.3079 |
| Q | 1.1486 | 1.3599 |
| N | 1.0976 | 1.3140 |
| S | 0.9132 | 0.9780 |
| T | 0.9642 | 1.0511 |
| A | 0.7344 | 0.6155 |
| I | 0.9587 | 0.7000 |
| L | 0.7196 | 0.5568 |
| V | 0.7698 | 0.5525 |
| F | 0.7910 | 0.6149 |
| P | 1.0588 | 1.2124 |
| G | 0.9514 | 0.9477 |
| M | 1.1952 | 0.9241 |
| C | 0.5261 | 0.3661 |

**Table S3**. Values of the features F1 and F2, defined as the ratio of amino acid frequencies in epitopes and the remaining antigen surface, and as the ratio of amino acid frequencies in epitopes and the remaining antigen, respectively.
