# Supplementary material for: SEPIa, a knowledge-driven algorithm for predicting conformational B-cell epitopes from the amino acid sequence
Source: BMC Bioinformatics. 2017 Feb 10;18:95. doi: 10.1186/s12859-017-1528-9 (PMC5301386; doi:10.1186/s12859-017-1528-9)
Supplement: Additional file 4: — SEPIa prediction model, implemented using the scikit-learn package. (ZIP 3901 kb) [file 12859_2017_1528_MOESM4_ESM.zip › SEPIa/SEPIa_soft.tar!/SEPIa_soft/Features/Amino_acid_composition.docx]

| Amino acid | Feature F1 | Feature F2 |
| --- | --- | --- |
| W | 1.2344 | 1.0960 |
| Y | 1.3511 | 1.2698 |
| H | 1.2063 | 1.4022 |
| K | 1.2056 | 1.5608 |
| R | 1.1327 | 1.3991 |
| D | 1.2141 | 1.4334 |
| E | 1.0320 | 1.3079 |
| Q | 1.1486 | 1.3599 |
| N | 1.0976 | 1.3140 |
| S | 0.9132 | 0.9780 |
| T | 0.9642 | 1.0511 |
| A | 0.7344 | 0.6155 |
| I | 0.9587 | 0.7000 |
| L | 0.7196 | 0.5568 |
| V | 0.7698 | 0.5525 |
| F | 0.7910 | 0.6149 |
| P | 1.0588 | 1.2124 |
| G | 0.9514 | 0.9477 |
| M | 1.1952 | 0.9241 |
| C | 0.5261 | 0.3661 |

Use the amino acid frequencies for every residue in the sequence.

Value of the features F1 and F2, defined as the ratio of amino acid frequencies in epitopes and the remaining antigen surface, and ratio of amino acid frequencies in epitopes and the remaining antigen, respectively.
