# Supplementary material for: SEPIa, a knowledge-driven algorithm for predicting conformational B-cell epitopes from the amino acid sequence
Source: BMC Bioinformatics. 2017 Feb 10;18:95. doi: 10.1186/s12859-017-1528-9 (PMC5301386; doi:10.1186/s12859-017-1528-9)
Supplement: Additional file 4: — SEPIa prediction model, implemented using the scikit-learn package. (ZIP 3901 kb) [file 12859_2017_1528_MOESM4_ESM.zip › SEPIa/SEPIa_soft.tar!/SEPIa_soft/Features/Hydrophilicity.docx]

| Amino acid | hydrophilicity |
| --- | --- |
| F | -2.5 |
| M | -1.3 |
| I | -1.8 |
| L | -1.8 |
| V | -1.5 |
| C | -1 |
| W | -3.4 |
| A | -0.5 |
| T | -0.4 |
| G | 0 |
| S | 0.3 |
| P | 0 |
| Y | -2.3 |
| H | -0.5 |
| Q | 0.2 |
| N | 0.2 |
| E | 3 |
| K | 3 |
| D | 3 |
| R | 3 |

Use the amino acid frequencies for every residue in the sequence.

hydrophilicity scale of Hopp and Woods
